# Predatory Marriage: An Emerging Medicolegal Issue

**DOI:** 10.1177/07067437231182564

**Published:** 2023-06-13

**Authors:** Julia G. Kirkham, Albert H. Oosterhoff, Kim A. Whaley, Freshta Akbary, Kenneth I. Shulman

**Affiliations:** 1 Department of Psychiatry, Cumming School of Medicine, 2129 University of Calgary, Calgary, AB, Canada; 2 6221 Faculty of Law, Western University, London, ON, Canada; 3 Whaley Estate Litigation (WEL) Partners, Toronto, ON, Canada; 4 Department of Psychiatry, 71545 Sunnybrook Health Sciences Centre, University of Toronto, Toronto, Ontario, Canada

**Keywords:** decision-making capacity, medicolegal issues, capacity to marry, adult psychiatry, geriatric psychiatry, cognitive deficits, developmental disabilities

Predatory marriage is a form of financial abuse and refers to marriage for the purpose of exploitation or personal profit—usually to gain control of a spouse's finances and/or receive that person's property after death.^1,2^ The victim is commonly an individual with impaired capacity to make a choice to marry, typically as a result of cognitive impairment or a psychiatric disorder. While the capacity to marry remains a legal determination, the conditions that increase vulnerability to predatory marriages are often within the purview of, or have implications for, psychiatric practice.

Determining the prevalence of predatory marriages is difficult due to the often covert nature of these relationships and the vulnerabilities of the victims. Predators typically isolate a victim or conceal a marriage from those who may question their intent. Predatory marriages can come to light in court challenges during the lifetime of the victim or after death. Such legal challenges used to be rare but are increasing in Canada and elsewhere, suggesting that predatory marriage is becoming more common.

The number of seniors has more than tripled in Canada in the past 40 years with those aged 75+ growing at the fastest rate; many more individuals with higher net worth will die in the coming decade than in previous ones.^3^ Greater wealth provides the incentive for would-be predators motivated by financial gain. At the same time, the population of those at risk, due to conditions that may impair capacity in later life, especially dementia, has also increased. Other factors that may be related to aging or psychiatric illness, such as disability and social isolation, may also raise the risk of victimization by marriage. Predatory marriages commonly involve an older adult, but not always, and may involve a victim with any condition that increases the risk of diminished capacity for decision making, transiently or permanently. These conditions include psychiatric disorders and cognitive impairment arising from neurodegenerative disease, intellectual disability, or acquired brain injury.

Capacity for any decision making involves two components: the ability to *understand* the information relevant to a decision and to *appreciate* the risks and benefits of a decision or lack of decision. All decision-making capacities including the capacity to marry are task/decision, time- and situation-specific.^2,4^ Practically, the latter means that in a simple situation with minimal risks, a lower level of understanding may suffice, whereas in a more complicated situation, a higher level is required.^4^ The legal standard for the capacity to marry has historically been a low one. The requisite capacity to marry has been considered equivalent to the capacity to enter into a contract, needing only an understanding of the nature of the contract (i.e., the relationship) and the responsibilities it involves.^1^ The typical standard, established in common law more than 100 years ago, is considered a “simple” test for which the necessary abilities are minimal, reflecting the relatively simple nature of marriage and its property implications a century ago.^1^ In *Wolfman-Stotland*, the court indicated that the capacity to marry or divorce requires “the lowest level of understanding,” less than that of the capacity to manage property or make a Will.^5^ The courts have indicated that the understanding required includes that marriage will have an effect on one's future, is monogamous (legally), and involves mutual support and cohabitation until death or divorce.^6^ Like other decisional capacities, the capacity to marry is specific to an individual's circumstances—marriage and the responsibilities arising from it will differ among couples.^2,4^ Separation and divorce are viewed by the courts as similar to the act of marriage with the same low standard extending to both capacities.

These legal thresholds do not reflect the complexities, both familial and economic, of modern-day relationships. Many people now divorce, have multiple marriages, children, and step-children, across their lifespan. The goals of marriage and the roles within it have also changed substantially over the past century. Marriage now comes with significant financial risk for the individual marrying as well as the potential beneficiaries of their estate. Some courts in Canada and abroad have recently applied a higher threshold, in which the ability to independently manage one's financial affairs is inherent to a person's understanding of the marriage contract. The higher threshold, however, has not been applied consistently and some cases continue to be decided based on a low standard, where the capacity to marry exists despite incapacity in other areas, including for managing property.^7^ Effectively, this means that many predatory marriages are very challenging to overturn in the courts, even when there is clear evidence of predatory intent. The financial and other risks that marriage now entails have led to calls for the standard of capacity to marry to be more stringent and require (in addition to the usual criteria) an appreciation of the effect of marriage on property and relationships, as well as the absence of undue influence. Changes to property and estate law could also offer protections from the financial consequences of predatory marriage, such as recent legislation in Ontario repealing a previous provision that revoked a prior Will upon marriage.

In most Canadian jurisdictions, legislation requires that individuals have the capacity to consent to marriage before a marriage license is issued or a ceremony performed but does not specify what the requisite capacity is or how it is determined. It is practically difficult to prevent a marriage since there is no clear process for determining capacity when a marriage licence is granted^1^ and since predators often hide their intentions or isolate the victim, such that there is rarely knowledge of a marriage in advance. Assessments of the capacity to marry (or separate and divorce) are still uncommon in clinical practice but may become more frequent. While there may not be an obligation to conduct a nontreatment-related capacity assessment, psychiatrists may wish to consider whether it is in their patient's best interests to do so and whether there are clinical, legal or ethical responsibilities in possible predatory relationships.^8^ Importantly, it is necessary to be cautious in identifying the reasonable cause for concern and biased perspectives. Ageist assumptions or stigma, for example, that those with mental illness or older people are undesirable as a result of disability or frailty, often drive the assumption that a would-be spouse's intentions must be predacious.^9^ The level of potential risk must be substantial enough to question an individual's capacity. Potentially removing or questioning an individual's rights to decide to marry should be viewed as a significant infringement on their autonomy; many individuals with mental illness or cognitive impairment retain the capacity to make a decision to marry. Regardless of whether the capacity to marry has been formally assessed, physicians should be aware that their medical records often become evidence in legal challenges.

Being a victim of predatory marriage results in significant financial loss, among other consequences, both in life and after death. More consistent legal protections are needed across Canada to mitigate the risk of financial and other harm from predatory marriages involving vulnerable individuals. Until then, psychiatrists may have an important role in protecting those at risk of victimization by predatory marriage and in supporting the autonomy of capable individuals.
